# Electro-Thermal Improvement in a β-Ga_2_O_3_ Cage-Integrated Slanted-Fin MOSFET

**DOI:** 10.3390/mi17050590

**Published:** 2026-05-11

**Authors:** Jianing Li, Yuan Li, Kai Peng, Xiaoli Lu, Xiaohua Ma

**Affiliations:** The State Key Laboratory of Wide-Bandgap Semiconductor Devices and Integrated Technology, Xidian University, Xi’an 710071, China; 21111110504@stu.xidian.edu.cn (J.L.); 23111213670@stu.xidian.edu.cn (K.P.); xhma@xidian.edu.cn (X.M.)

**Keywords:** β-Ga_2_O_3_ MOSFET, electric-field management, self-heating, electro-thermal improvement

## Abstract

Electro-thermal improvement is critical for β-Ga_2_O_3_ power devices to mitigate self-heating while maintaining high-voltage capability. Here, we propose a β-Ga_2_O_3_ cage-integrated slanted-fin MOSFET (C-SFMOSFET). By optimizing the cage-to-fin and cage-to-drain distances, the cage sequence simultaneously strengthens channel depletion and enhances heat dissipation in the gate-to-drain region. Compared with the baseline slanted-fin MOSFET (SFMOSFET), the proposed 4-cage C-SFMOSFET achieves a 1.75× higher Baliga’s figure of merit and reduces the peak junction temperature by 8 °C at 0.55 W/mm. These results indicate that the proposed device layout can effectively improve device-level electro-thermal performance and further exploit the inherent advantages of ultra-wide-bandgap β-Ga_2_O_3_.

## 1. Introduction

The ultra-wide-bandgap (UWBG) semiconductor β-Ga_2_O_3_ has attracted considerable research interest for next-generation power devices because of its high theoretical Baliga’s figure of merit (BFOM), its large breakdown electric field, and the availability of large-size substrates [1,2,3,4,5]. These advantages make β-Ga_2_O_3_ a promising candidate for high-power and high-efficiency switching applications.

These material properties also broaden the device-level design space for electric-field engineering [6,7]. For example, Wang et al. demonstrated a lateral superjunction-equivalent MOSFET [8], which achieved full depletion and punch-through operation as the electric field approached the critical value of β-Ga_2_O_3_. However, selective p-NiO filling in the drift region degraded the on-state performance. Meng et al. introduced a self-aligned vertical gate structure together with a gate field plate into a lateral MOSFET, reducing the electric field in the gate-to-drain access region and suppressing nonlinear current rise in saturation [9]. However, the stringent process requirements of the self-aligned vertical gate indicate that electrostatic optimization may come with manufacturability penalties. These studies show that electrical-performance enhancement in β-Ga_2_O_3_ MOSFETs is feasible but often involves practical or structural trade-offs.

More critically, the relatively low thermal conductivity of β-Ga_2_O_3_ (10.9–27 W/m·K at 300 K) makes self-heating a persistent bottleneck, so that even effective electric-field engineering remains thermally constrained [10]. To address this issue, several thermal-management approaches have been explored. Substrate-level strategies, such as substrate thinning and heterogeneous integration with high-thermal-conductivity materials, can reduce the thermal resistance of β-Ga_2_O_3_ MOSFETs [11,12,13]. However, the mechanical fragility of β-Ga_2_O_3_ complicates aggressive thinning and may introduce extra cost and reliability concerns. Layout-oriented studies have also shown that topside geometry strongly influences heat extraction. For example, Kim et al. reported that reducing the gate-to-drain distance in lateral β-Ga_2_O_3_ transistors lowered the peak temperature through enhanced heat extraction by the drain electrode, but at the expense of breakdown voltage [14]. They further showed that channel orientation and drain-interconnection geometry also play important roles in thermal management [15]. In addition, thermal characterization studies, such as Raman thermography of β-Ga_2_O_3_ fin-based devices, have highlighted the importance of thermal management and the associated electro–thermal trade-offs [16]. Collectively, these studies indicate that prior efforts mainly focused on electric-field optimization, thermal management, or identifying electro-thermal trade-offs, but did not establish a practical device-layout-level strategy to mitigate this trade-off in a unified manner.

These limitations motivate device-level electro-thermal co-optimization in β-Ga_2_O_3_ power devices. In our previous work, a β-Ga_2_O_3_ slanted-fin MOSFET (SFMOSFET) was proposed to reshape the electrostatic potential profile beneath the gate and promote a more favorable drain-side extension of the depletion region, thereby improving electric-field management and BFOM compared with a straight-fin device [17]. However, although slanted-fin geometry enhances depletion modulation under the gate, electric-field crowding and thermal accumulation in the gate-to-drain region remain significant challenges. More importantly, once additional field-modulation elements are introduced, their interaction with the slanted-fin-induced depletion profile becomes a nontrivial co-design issue rather than a simple structural add-on. In other words, the placement of a floating cage sequence in a slanted-fin MOSFET should be coordinated with the slanted-fin-induced depletion boundary, so that the favorable gate-controlled depletion can be preserved while the remaining gate-to-drain region is further regulated. Meanwhile, the cage sequence can provide an additional topside heat-spreading path.

Unlike a simple extension of our previous work, the present study focuses on the co-design between the slanted-fin-induced depletion profile and the floating cage sequence for simultaneous electric-field and thermal improvement. Based on this concept, this study proposes a β-Ga_2_O_3_ cage-integrated slanted-fin MOSFET (C-SFMOSFET). By jointly optimizing the cage-to-fin distance and cage-to-drain distance, the proposed device is designed to alleviate electric-field crowding and self-heating in the gate-to-drain region in a unified manner. Comprehensive electro-thermal simulations and experimental characterization verify the advantages of the C-SFMOSFET and provide a device-layout-level electro-thermal design strategy for thermally constrained β-Ga_2_O_3_ power devices. The novelty does not lie in the use of metal stripes alone, but in their coordinated layout with the slanted-fin-induced depletion boundary to achieve device-level electro-thermal co-optimization.

## 2. Cage Sequence Design by Electro-Thermal Analysis

### 2.1. Device Structure and Modeling Framework

This section summarizes the co-simulation workflow, device dimensions, and thermal material parameters used in the following electro-thermal analysis. The proposed C-SFMOSFET is derived from the baseline SFMOSFET and evaluated using a two-step framework combining Sentaurus (O-2018.06-SP2) and ANSYS Icepak (2019 R3).

Sentaurus was first used to construct the device models and simulate device operation under specified bias conditions. From these simulations, the electrostatic potential, electric-field distribution, depletion profile, and internal temperature distribution were obtained to identify the active heat-generation region. ANSYS Icepak was then employed to evaluate topside heat dissipation to the surrounding air for different device layouts. In Icepak, an equivalent heat-source distribution was assigned to the active β-Ga_2_O_3_ region, according to the heat-generation region identified from the Sentaurus simulation, and the same total dissipated power was applied to all layouts to ensure a fair comparison. Steady-state thermal fluid analysis was performed with coupled flow and temperature fields. Natural convection was taken into account by enabling gravity, and all six faces of the surrounding air domain were defined as opening boundaries. The resulting air-velocity distributions above the device were then compared to assess the relative topside heat-dissipation capability of different layouts.

**Figure 1:**
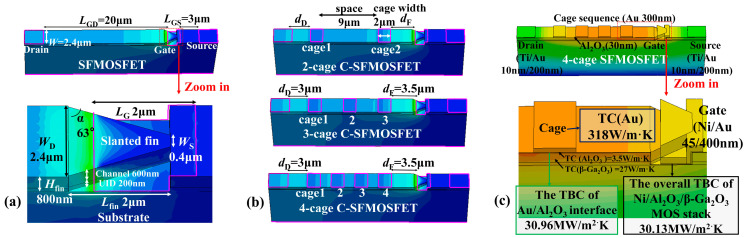
(**a**) Schematic structure of the SFMOSFET and zoomed-in view of the slanted-fin region. (**b**) Topological structures of the 2-cage, 3-cage, and 4-cage C-SFMOSFETs with the proper cage-boundary position (*d*_D_ = 3 μm and *d*_F_ = 3.5 μm). (**c**) Thermal material parameters used in the electro-thermal device models.

The SFMOSFET employed the following geometrical parameters (Figure 1a): gate-to-drain length *L*_GD_ = 20 μm, gate length *L*_G_ = 2 μm, gate-to-source length *L*_GS_ = 3 μm, and drain-to-source length *L*_DS_ = 25 μm. The slanted fin structure had the following parameters: drain-side width *W*_D_ = 2.4 μm, source-side width *W*_S_ = 0.4 μm, fin angle α = 63°, fin length *L*_fin_ = 2 μm, and fin height *H*_fin_ = 800 nm. The gate width is *W*_G_ = 2 μm [18] and the gate dielectric is 30-nm-thick Al_2_O_3_.

The thermal parameters used in the model are summarized in Figure 1c. The thermal conductivities were taken from the literature: β-Ga_2_O_3_ = 27 W/m·K [19], Al_2_O_3_ = 3.5 W/m·K [20], Ti = 180 W/m·K [21], Ni = 90 W/m·K [21], and Au = 318 W/m·K [21]. The remaining interface-related parameters were obtained via time-domain thermoreflectance (TDTR) [22]. For the Ni/Al_2_O_3_/β-Ga_2_O_3_ (45 nm/30 nm/500 μm) sample, the overall thermal boundary conductance (TBC) of the MOS stack was measured to be 30.13 MW/m^2^·K. Au was selected as the cage material for electro-thermal evaluation, although other materials may offer similar electro-thermal behavior [23]. For the Au/Al_2_O_3_/β-Ga_2_O_3_ (300 nm/30 nm/500 μm) sample, the TBC at the Au/Al_2_O_3_ interface was 30.96 MW/m^2^·K.

### 2.2. Electric-Field Optimization of the Cage Sequence

Because the gate-to-drain spacing of the SFMOSFET exceeds its maximum depletion width, an electrically floating metal cage sequence was introduced to further regulate the electrostatic potential in the gate-to-drain region. The cage sequence interacts strongly with the slanted-fin-induced depletion extension. Hence, its boundary position should be coordinated with the slanted-fin-induced depletion region; otherwise, the favorable depletion regulation established by the slanted-fin may be compromised. The optimization, therefore, proceeded in two steps: boundary-position optimization first, followed by cage-geometry optimization.

**Figure 2:**
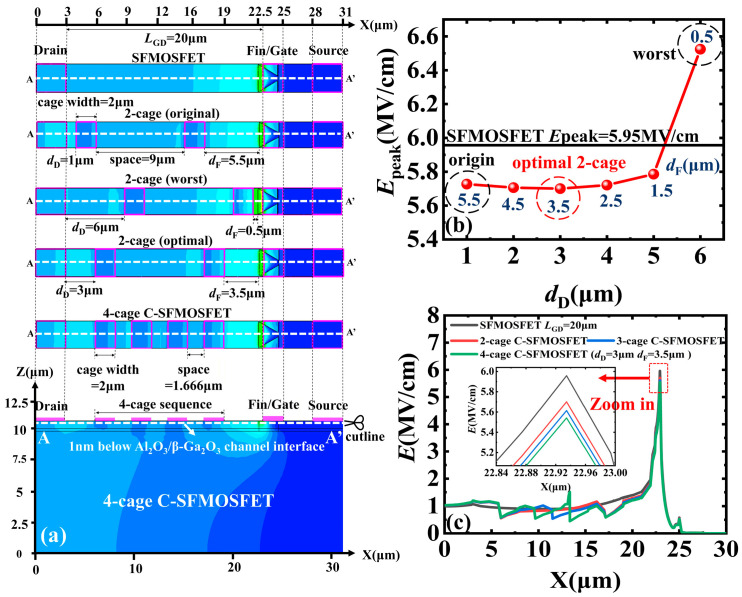
(**a**) Top-view layouts of the SFMOSFET, original 2-cage (*d*_D_ = 1 μm, *d*_F_ = 5.5 μm), worst 2-cage (*d*_D_ = 1 μm, *d*_F_ = 0.5 μm), optimal 2-cage (*d*_D_ = 3 μm, *d*_F_ = 3.5 μm), and proposed 4-cage C-SFMOSFET (*d*_D_ = 3 μm, *d*_F_ = 3.5 μm), together with the cutline A–A′, located 1 nm below the Al_2_O_3_/β-Ga_2_O_3_ channel interface. (**b**) Extracted *E*_peak_ of 2-cage C-SFMOSFET along the cutline A–A′ for different cage-sequence positions, represented by varied *d*_D_ and *d*_F_. (**c**) Off-state electric-field profiles of SFMOSFET, 2-cage, 3-cage, and 4-cage C-SFMOSFETs along the cutline A–A′ at *V*_GS_ = −15 V and *V*_DS_ = 1000 V.

A 2-cage sequence was first used to determine the proper boundary position. In this design, each cage width was 2 μm and the inter-cage spacing was 9 μm within *L*_GD_ = 20 μm. The 2-cage sequence was shifted to different locations as a whole unit along the gate-to-drain direction of the SFMOSFET, while keeping the cage width and inter-cage spacing fixed. Here, *d*_D_ denotes the distance from the cage edge to the drain and *d*_F_ denotes the distance from the cage edge to the fin (Figure 2a). After boundary optimization, a 4-cage C-SFMOSFET with the same overall device dimensions was constructed for cage-number evaluation, as shown in Figure 1b.

The modulated mechanism involves electrostatic potential redistribution among adjacent floating cages. In the current work, the off-state condition refers to the bias state in which the device is turned off while a high drain voltage is applied (e.g., *V*_GS_ = −15 V and *V*_DS_ = 1000 V, unless otherwise specified). The electrostatic potential difference between neighboring cages is described by Formula (1) [24], where *ϕ_i_* is the electrostatic potential of the cage i, *Q*_i_ is the induced charge of cage i, and *C*_i_ is the capacitance between adjacent cage i and cage i + 1. The cage closest to the drain electrode is defined as cage 1. Accordingly, *ϕ*_0_ denotes the electrostatic potential of the drain electrode, and *W*_depletion_ denotes the depletion-region width. As indicated by Formula (2), extending depletion reduces the lateral gate-edge off-state electric-field peak (*E*_peak_). Distributing the electrostatic potential across multiple cages, therefore, smooths the off-state electric-field profile in the slanted-fin and cage region and improves breakdown voltage (*V*_BR_).

(1) $$\phi_{i}-\phi_{i+1}=\frac{Q_{i}}{C_{i}}$$

(2) $$E_{\mathrm{peak}}=\frac{V_{\mathrm{Drain}}-\sum_{i=0}^{n-1}(\phi_{i}-\phi_{i+1})}{W_{\mathrm{depletion}}}$$

**Figure 3:**
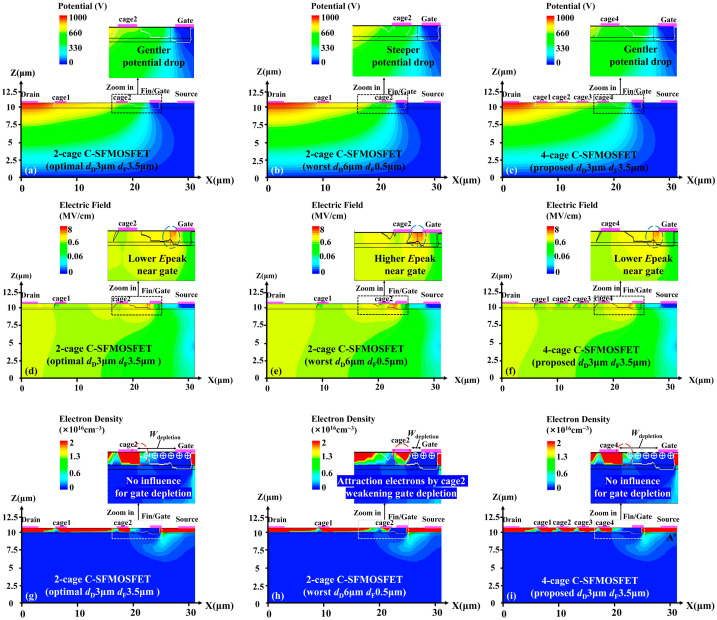
Simulated full-device distributions of: (**a**–**c**) electrostatic potential, (**d**–**f**) off-state electric-field, and (**g**–**i**) the depletion region of the optimal 2-cage C-SFMOSFET, worst 2-cage C-SFMOSFET, and proposed 4-cage C-SFMOSFET at *V*_GS_ = −15 V and *V*_DS_ = 1000 V. Insets show zoomed-in views of the gate-edge region. The circled plus symbols schematically denote ionized donors, and the areas filled with these symbols represent the depletion region.

The boundary optimization process follows a clear design rule: too small a *d*_F_ should be avoided, because the attractive effect of the cage on channel electrons perturbs the favorable depletion extension induced by the slanted-fin structure and thereby weakens the original gate-controlled depletion, resulting in a higher *E*_peak_ (Figure 3b,e,h). In the 2-cage C-SFMOSFET, the minimum *E*_peak_ of 5.7 MV/cm at the off-state bias *V*_GS_ = −15 V and *V*_DS_ = 1000 V is obtained at *d*_D_ = 3 μm and *d*_F_ = 3.5 μm (Figure 2b). These optimized boundary parameters were then used for the subsequent cage-number optimization. Based on this optimized boundary, the 4-cage C-SFMOSFET further reduces off-state *E*_peak_ by 7%, from 5.96 MV/cm in the SFMOSFET to 5.54 MV/cm under the same bias condition (Figure 2c).

**Figure 4:**
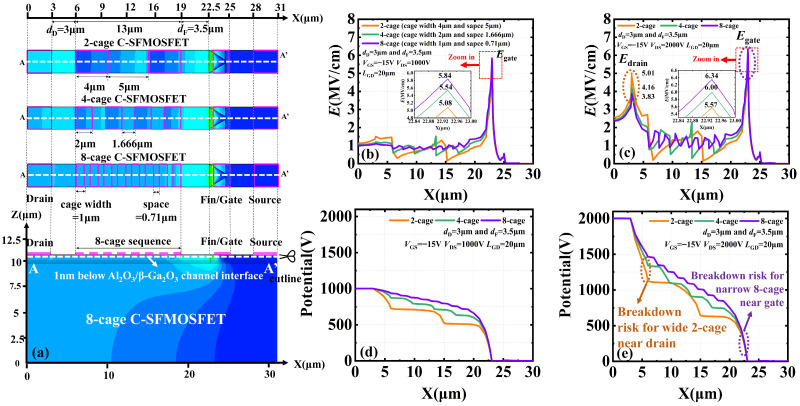
(**a**) Top-view layouts of the 2-cage (wider), 4-cage (proposed), and 8-cage (narrower) sequences, together with cutline A–A′ located 1 nm below the Al_2_O_3_/β-Ga_2_O_3_ channel interface. Off-state electric-field profiles of the 2-cage, 4-cage, and 8-cage sequences are given along the cutline A–A′ (**b**) at *V*_GS_ = −15 V and *V*_DS_ = 1000 V and (**c**) at *V*_GS_ = −15 V and *V*_DS_ = 2000 V. Corresponding electrostatic potential profiles are given along the cutline A–A′ (**d**) at *V*_GS_ = −15 V and *V*_DS_ = 1000 V and (**e**) at *V*_GS_ = −15 V and *V*_DS_ = 2000 V.

The optimum cage geometry parameters should be selected to achieve the best overall electric-field balance in the gate-to-drain region. To this end, 2-cage (wider), 4-cage (proposed), and 8-cage (narrower) sequences were compared, using the same proper cage-boundary positions (*d*_D_ = 3 μm and *d*_F_ = 3.5 μm) and the same total cage area. As shown in Figure 4a, the individual cage widths were 4, 2, and 1 μm for the 2-cage, 4-cage, and 8-cage sequences, respectively. At the off-state bias *of V*_GS_ = −15 V and *V*_DS_ = 1000 V, the simulated gate-edge electric-field peaks (*E*_gate_ or *E*_peak_) were 5.08, 5.54, and 5.84 MV/cm, respectively (Figure 4b). The 2-cage sequence exhibited the lowest *E*_gate_ at this bias because its wider cages provided stronger capacitive coupling to the channel and, thus, stronger electrostatic potential modulation, even with a smaller cage number (Figure 4d). However, this stronger modulation also produced a larger electrostatic potential drop across the cage-controlled region, which shifted the electric-field concentration toward the drain edge.

This trade-off became more evident at a higher drain bias under off-state blocking conditions. At *V*_GS_ = −15 V and *V*_DS_ = 2000 V, *E*_gate_ increased to 5.57, 6.00, and 6.34 MV/cm for the 2-cage, 4-cage, and 8-cage sequences, respectively, while the corresponding drain-side electric-field peaks (*E*_drain_) were 5.01, 4.16, and 3.83 MV/cm (Figure 4c). The higher *E*_drain_ in the 2-cage sequence indicates that excessively wide cages, despite their strong capacitive coupling to the channel, tend to transfer the high-electric-field region toward the drain edge and, thus, increase the risk of premature breakdown [3]. In contrast, excessively narrow cages in the 8-cage sequence weaken the local capacitive coupling to the channel. As a result, although the cage number is larger, the local electrostatic potential modulation remains weak, the electrostatic potential drop near the gate side becomes steeper, and *E*_gate_ correspondingly increases (Figure 4e). Therefore, cage geometry should be optimized by balancing the stronger drain-edge electric field induced by overly wide cages against the higher gate-edge electric field caused by overly narrow cages. On this basis, the 4-cage sequence provides the best overall electric-field balance and was therefore adopted in this work.

### 2.3. Electric-Field Modulation After Device Scaling

**Figure 5:**
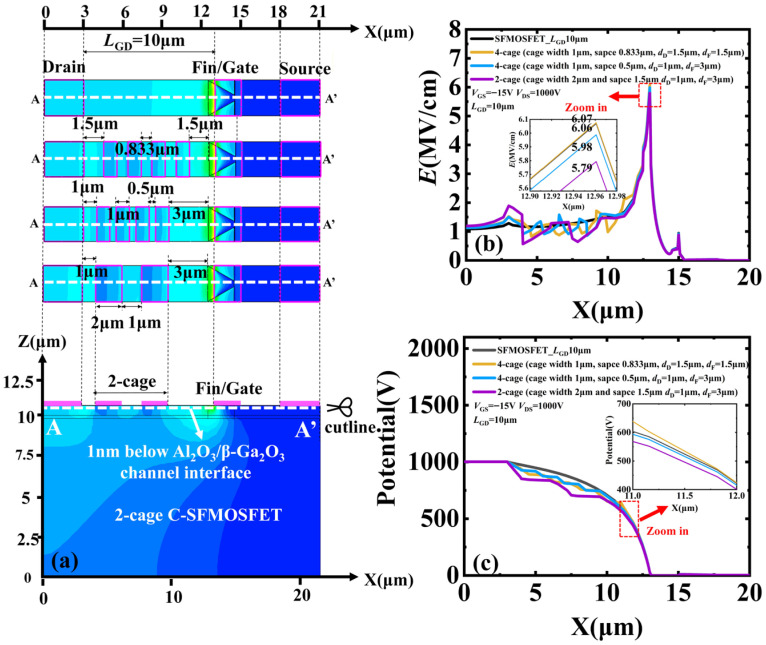
(**a**) Top-view layouts of the SFMOSFET and three scaled cage-design layouts within *L*_GD_ = 10 μm, together with the cutline A–A′ located 1 nm below the Al_2_O_3_/β-Ga_2_O_3_ channel interface. (**b**) Off-state electric-field profiles along the cutline A–A′ at *V*_GS_ = −15 V and *V*_DS_ = 1000 V. (**c**) Corresponding electrostatic potential profiles along the cutline A–A′ at *V*_GS_ = −15 V and *V*_DS_ = 1000 V.

The design principle remained valid after device scaling, but an effective cage sequence still required proper co-design with the slanted-fin-induced depletion extension, together with re-optimization of the cage width to maintain strong electrostatic potential modulation. To verify this, the gate-to-drain spacing was reduced from 20 to 10 μm, and three scaled layouts were compared with the reference SFMOSFET, as shown in Figure 5a. The first scaled 4-cage layout was obtained by the nearly proportional scaling of the original proposed 4-cage design, with the cage width reduced from 2 μm to 1 μm and the boundary distances reduced from *d*_D_ = 3 μm and *d*_F_ = 3.5 μm to *d*_D_ = 1.5 μm and *d*_F_ = 1.5 μm. The second scaled 4-cage layout retained the 1 μm cage width but used *d*_D_ = 1 μm and *d*_F_ = 3 μm to preserve a wider *d*_F_ for the favorable depletion extension induced by the slanted-fin structure. Based on this cage-boundary-preserved layout, the third scaled 2-cage layout further increased the cage width to 2 μm, while keeping *d*_D_ = 1 μm *and d*_F_ = 3 μm in order to strengthen capacitive coupling to the channel.

At the off-state bias of *V*_GS_ = −15 V and *V*_DS_ = 1000 V, the *E*_gate_ of the reference SFMOSFET and the three scaled layouts are 6.06, 6.07, 5.98, and 5.79 MV/cm, as shown in Figure 5b, respectively. Nearly proportional scaling provided almost no improvement, because the reduced *d*_F_ places the cage sequence too close to the slanted-fin gate and weakens the favorable depletion extension. In contrast, preserving sufficient cage-boundary spacing restored the intended depletion-assisted modulation, while further increasing the cage width enhances capacitive coupling and improves electrostatic potential redistribution, as shown in Figure 5c. These results indicate that, after scaling, effective cage optimization depends on both proper cage-boundary matching and an adequate cage width within the reduced gate-to-drain design.

### 2.4. Enhanced Heat Dissipation Considering Self-Heating

In addition to the electric-field benefits discussed above, the cage sequence also influences the thermal behavior of the device. The superior thermal performance of the proposed 4-cage C-SFMOSFET compared with the baseline SFMOSFET can be understood as the combined effect of enhanced upward heat spreading within the device and improved topside heat dissipation to the ambient air.

**Figure 6:**
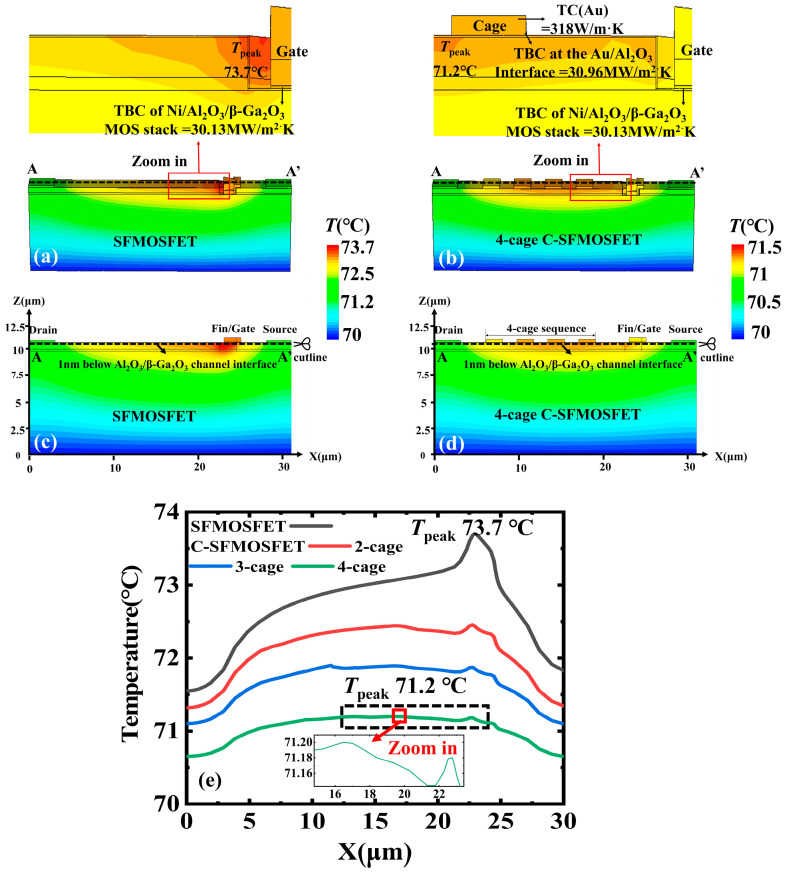
Temperature distributions of (**a**) SFMOSFET and (**b**) 4-cage C-SFMOSFET at *V*_GS_ = 10 V and *P* = 0.12 W/mm. Insets show zoomed-in views of the hotspot region. Cross-sectional temperature distributions are also shown of (**c**) SFMOSFET and (**d**) 4-cage C-SFMOSFET, together with the cutline A–A′ located 1 nm below the Al_2_O_3_/β-Ga_2_O_3_ channel interface. (**e**) The corresponding temperature profiles along the cutline A–A′ for the SFMOSFET and the 2-cage, 3-cage, and 4-cage C-SFMOSFETs.

In lateral β-Ga_2_O_3_ devices, the primary heat source is located in the channel region near the gate (Figure 6a–d). Owing to the TBC at the Au/Al_2_O_3_ interface of 30.96 MW/m^2^·K, part of the heat generated in the channel can be transferred upward to the Au cage layer, which provides an additional heat-spreading path [25]. This upward heat-extraction effect becomes more evident with increasing cage number (Figure 6e). Under identical power dissipation (*P* = 0.12 W/mm), the 4-cage C-SFMOSFET reduces the peak junction temperature (*T*_peak_) by 2.5 °C compared with the SFMOSFET.

In addition to improved internal heat-spreading, the 4-cage sequence also contributes to better topside heat dissipation by increasing the exposed surface area. Under natural-convection conditions, Formula (3) indicates that the convective heat transfer rate *Q*_conv_ is related to the convective heat transfer coefficient *h* and the surface area exposed to the fluid *A* [26].

(3) $$Q_{\mathrm{conv}}\propto hA$$

**Figure 7:**
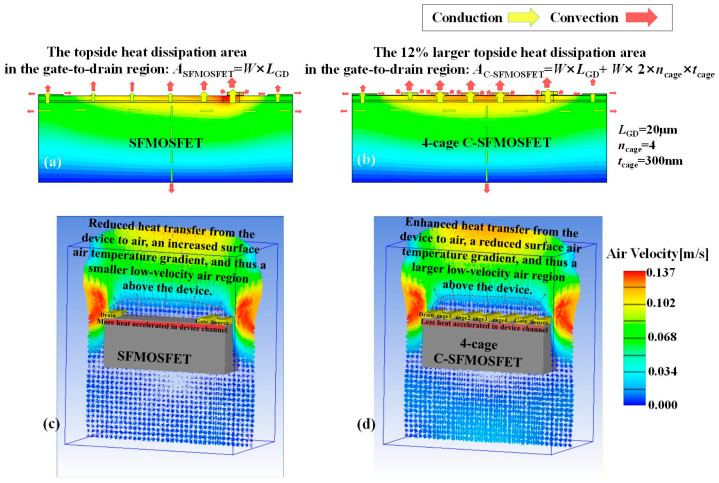
Schematic illustration of the heat-transfer pathways and topside heat-dissipation areas for (**a**) the SFMOSFET and (**b**) the 4-cage C-SFMOSFET under identical power dissipation (*P* = 0.12 W/mm). Air-velocity distributions are shown for (**c**) the SFMOSFET and (**d**) the 4-cage C-SFMOSFET under identical power dissipation (*P* = 0.12 W/mm). The dashed outlines in (**c**) and (**d**) indicate the boundaries of the low-air-velocity regions above the device.

Since the convective heat transfer coefficients at both Al_2_O_3_/air (for SFMOSFET) and Au/air interfaces (for 4-cage C-SFMOSFET) are primarily governed by the fluid (air) properties, a typical natural convection heat transfer coefficient of approximately 5 W/m^2^·K was adopted [27]. Consequently, the larger exposed surface area of the 4-cage C-SFMOSFET was expected to enhance topside convective heat dissipation under comparable natural-convection conditions. The disparity in natural convection heat dissipation performance between the SFMOSFET and 4-cage C-SFMOSFET was validated using the ANSYS Icepak simulation. The topside thermal pathway in the SFMOSFET was β-Ga_2_O_3_/Al_2_O_3_/air, whereas that in the 4-cage C-SFMOSFET was β-Ga_2_O_3_/Al_2_O_3_/cage/air. Benefiting from the 4-cage sequence with 300 nm-thick Au sidewalls, the 4-cage C-SFMOSFET provided a 12% larger topside heat dissipation area (Figure 7a,b). This led to enhanced heat transfer from the device to the surrounding air and alleviated local thermal accumulation in the near-surface ambient region, as reflected by the reduced temperature gradient near the surface and the larger low-air-velocity region above the device (Figure 7c,d) [28]. Therefore, the 4-cage C-SFMOSFET provides improved topside heat dissipation. This heat dissipation advantage offered by the 4-cage C-SFMOSFET becomes increasingly pronounced at higher power dissipation levels [11].

## 3. Experimental Verification and Discussion

### 3.1. Fabrication and Measurement

**Figure 8:**
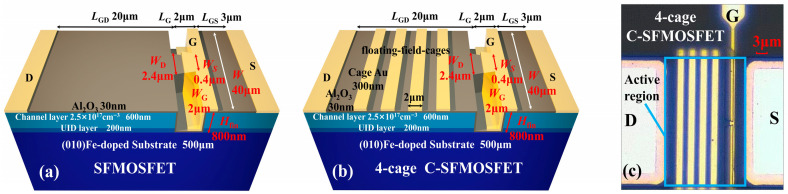
Schematic of the fabricated β-Ga_2_O_3_ (**a**) SFMOSFET and (**b**) 4-cage C-SFMOSFET. (**c**) Optical top-view image of the β-Ga_2_O_3_ C-SFMOSFET prototype. Colors in (**a**) and (**b**) are used to distinguish different material/structural layers, and the blue outline in (**c**) indicates the active region.

Both the 4-cage C-SFMOSFET and the SFMOSFET were fabricated for experimental validation (Figure 8a–c). A β-Ga_2_O_3_ epitaxial film with a channel layer (600 nm thick and with a 2.5 × 10^17^ cm^−3^ doping concentration) and an unintentionally doped layer (200 nm thick) was grown on an (010) Fe-doped semi-insulating substrate. Mesa isolation and a slanted-fin channel were etched to a depth of 800 nm via inductively coupled plasma (ICP) etching in a BCl_3_/Cl_2_ ambience. The slanted fin was defined by e-beam lithography, and surface damage was removed with piranha solution [29]. The gate oxide (30-nm-thick Al_2_O_3_), gate electrodes (45-nm/400-nm-thick Ni/Au), and source-drain electrodes (10 nm/200 nm-thick Ti/Au) were identical for both devices. The 4-cage sequence was formed by depositing 300-nm-thick, 2-μm-long Au layers along the device width on the SFMOSFET surface (Figure 8c).

Systematic electrical and thermal measurements were performed to investigate the electro-thermal performance of the 4-cage C-SFMOSFET and the SFMOSFET. Electrical characteristics, including output, transfer, and breakdown characteristics, were measured using a Keysight B1500A analyzer. In addition, I–V characteristics were measured under both DC and pulsed conditions [30]. The DC measurements included self-heating effects, while the pulsed measurements were performed with a duty cycle of 0.035% to suppress self-heating. This comparison was used to examine the current variation induced by self-heating in two devices qualitatively. The thermal performance was further characterized using a QFI InfraScope under identical power dissipation for quantitative analysis, with the ambient temperature set to 70 °C to obtain clear thermal images [31].

### 3.2. Experimental Results and Discussion

**Figure 9:**
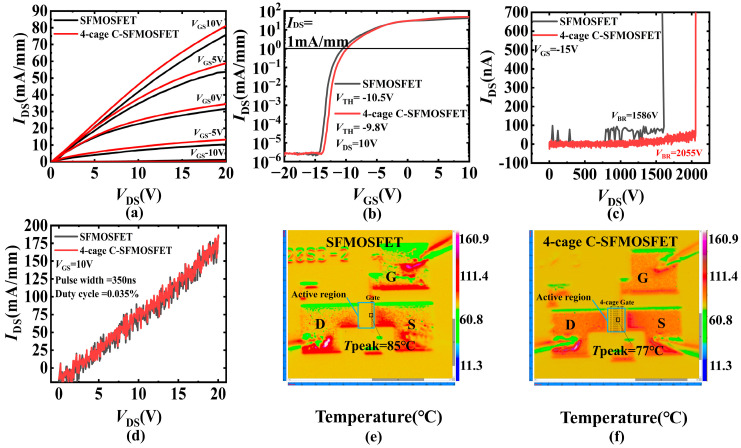
(**a**) Output characteristics of the DC measurement mode at various *V*_GS_ (10 V to −10 V, step of 5 V), (**b**) transfer characteristics at *V*_DS_ = 10 V (*V*_TH_ extracted at *I*_DS_ = 1 mA/mm), and (**c**) breakdown characteristics at *V*_GS_ = −15 V. (**d**) Pulsed measurement mode output characteristic at *V*_GS_ = 10 V. Steady-state thermal mapping images of (**e**) SFMOSFET and (**f**) 4-cage C-SFMOSFET at *P* = 0.55 W/mm. Solid boxes mark the active region corresponding to the fabricated device shown in Figure 8c, and dashed boxes indicate the gate region and the 4-cage sequence above the active region.

The 4-cage C-SFMOSFET prototype exhibited superior electro-thermal performance compared with the control SFMOSFET (Figure 9a–f and Table 1). The breakdown voltage (*V*_BR_) increased by 29% from 1586 V to 2055 V, and BFOM, defined as *V*_BR_^2^/*R*_on,sp_, improved by a factor of 1.75, where *R*_on,sp_ is the specific on-resistance value normalized to the active region area *W* × *L*_DS_. The threshold voltage (*V*_TH_), extracted at *I*_DS_ = 1 mA/mm for consistent comparison, shifted positively by 0.7 V. At *V*_GS_ = 10 V and *V*_DS_ = 20 V, the DC output current (considering the self-heating effect) increased by 7%, from 75.8 mA/mm to 81.1 mA/mm and the on-state resistance decreased by 4.3%, while the pulsed current showed no obvious change. The *T*_peak_ of the 4-cage C-SFMOSFET (77 °C) was 8 °C lower than that of SFMOSFET (85 °C) at *P* = 0.55 W/mm. The increased *V*_BR_ and the positive shift in *V*_TH_ are consistent with the electrostatic potential modulation and enhanced channel depletion introduced by the optimized cage sequence. The improved DC output current and reduced on-state resistance are associated with improved carrier transport resulting from alleviated self-heating.

This work experimentally validates the electro-thermal co-optimization of the cage sequence and slanted-fin-induced depletion. To better position the present work with respect to the existing literature, representative prior β-Ga_2_O_3_ MOSFET studies on electric-field control and thermal management are summarized in Table 2. As shown, these prior studies mainly focused on either electric-field engineering or thermal management, revealing important electrical–thermal trade-offs or improving only one aspect at a time, but not providing an experimentally validated device-layout-level strategy for simultaneous electro-thermal improvement. The present results support the proposed co-design strategy, in which the floating cage sequence is coordinated with the slanted-fin-induced depletion profile to further regulate the gate-to-drain region while also enhancing topside heat spreading.

## 4. Conclusions

In summary, an electro-thermally improved β-Ga_2_O_3_ C-SFMOSFET is presented in this study. The cage-integrated slanted-fin structure improves channel depletion and raises the breakdown voltage. In addition, the cage sequence promotes thermal transport and topside heat dissipation, which helps improve its high-temperature power handling. Despite the low thermal conductivity of β-Ga_2_O_3_, the proposed device-level electro-thermal improvement layout offers a practical balance between thermal constraints and electrical performance, providing useful design guidance for thermally sensitive β-Ga_2_O_3_ devices. Future work will extend this framework to the design of large-sized β-Ga_2_O_3_ power devices.

## Figures and Tables

**Table 1:** Measured performance comparison between SFMOSFET and 4-cage C-SFMOSFET.

Device	*I*_DS-DC_ at *V*_GS_ = 10 V *V*_DS_ = 20 V	*I*_DS-Pulse_ at *V*_GS_ = 10 V *V*_DS_ = 20 V	*R*_on_ at *V*_GS_ = 10 V	*V*_TH_ at *V*_DS_ = 10 V *I*_DS_ = 1 mA/mm	*V*_BR_ at *V*_GS_ = −15 V	BFOM	*T*_peak_ at *P* = 0.55 W/mm
Normalized by	*W*_G_	*W*_G_	*W*_G_	/	/	*W* × *L*_DS_	*W*
SFMOSFET (Control)	75.8 mA/mm	184.6 mA/mm	215.3 Ω·mm	−10.5 V	1586 V	2.34 MW/cm^2^	85 °C
4-cage C-SFMOSFET (Prototype)	81.1 mA/mm	186.1 mA/mm	206 Ω·mm	−9.8 V	2055 V	4.10 MW/cm^2^	77 °C
Relative variation	+7%	+0.8%	−4.3%	+0.7 V	+29%	×1.75	−8 °C

**Table 2:** Comparison with representative prior β-Ga_2_O_3_ MOSFET studies on electric-field control and thermal management.

Reference	Device/Strategy	Main Focus	Representative Result	Main Limitation Relative to This Work
Wang et al., TED 2022 [8]	Superjunction-equivalent between the gate-to-drain region	Electric-field management	*V*_BR_ = 1362 V; 4.86× BFOM	Electrical optimization only; no thermal improvement
Kim et al., ITherm 2021 [14]	Drain-metal heat extraction by gate-to-drain spacing variation	Electro-thermal trade-off	~35% higher Δ*T*_peak_ when *L*_GD_ decreased from 11 μm to 1 μm at 0.8 W/mm	Trade-off identified, but no mitigation strategy
Kim et al., TED 2022 [15]	Thermally aware lateral layout by channel direction and drain-interconnection width	Layout-level thermal management	~10% lower Δ*T*_peak_ by orientation optimization; ~8% higher ΔTpeak with narrower interconnects at 1 W/mm	Thermal optimization only; no simultaneous *V*_BR_/BFOM improvement
Kim et al., TED 2023 [11]	Double-sided diamond cooling	External thermal management	~75% lower steady-state Δ*T*_peak_ at 4 W/mm	External cooling only; no device-layout optimization
Zhao et al., APL 2026 [16]	Raman thermography of multi-fin β-Ga_2_O_3_ FinFETs	Thermal crosstalk	Near-gate hotspot: 70.77 °C in multi-fin vs. 28.8 °C in single-fin devices at 4.7 mW per fin	Trade-off identified, but no mitigation strategy
Xu et al., IEDM 2019 [12]	Ga_2_O_3_-on-SiC MOSFETs by ion-cutting	Substrate-level thermal management	Up to 500 K; ~14% *R*_on_ degradation; ~600 V sustained	Substrate integration only; no device-layout optimization
This work	Cage-integrated slanted-fin MOSFET	Layout-level electro-thermal co-optimization	*V*_BR_ = 2055 V; 1.75× BFOM; ~53% lower Δ*T*_peak_ relative to ambient (−8 °C in *T*_peak_) at 0.55 W/mm	Experimentally validated electro–thermal co-improvement at the device-layout level

## Data Availability

Data are contained within the article. The data that support the findings of this study are available from the corresponding authors upon reasonable request.
